# Understanding clients’ and providers’ perspectives on the implementation of subcutaneous depot medroxyprogesterone acetate (DMPA-SC) for self-injection programming in Nigeria

**DOI:** 10.1136/bmjgh-2024-018763

**Published:** 2026-02-26

**Authors:** Sneha Challa, Madeline Griffith, Ayobambo Jegede, Aminat Tijani, Emily Himes, Ivan Idiodi, Chioma Okoli, Shakede Dimowo, Elizabeth Omoluabi, Jenny X Liu

**Affiliations:** 1 Institute for Health & Aging, University of California, San Francisco, San Francisco, California, USA; 2 AkenaPlus Health, Abuja, Nigeria; 3 University of California, San Francisco, San Francisco, California, USA

**Keywords:** Global Health, Qualitative study, Public Health

## Abstract

Subcutaneous depot medroxyprogesterone acetate (DMPA-SC) is an injectable contraceptive method with a small needle and prefilled syringe system that has been approved for self-injection (SI) by clients. As DMPA-SC for SI programmes are being scaled, employing an implementation science lens is critical to understanding what works. This study explored providers’ and clients’ experiences with providing and receiving services, respectively, for DMPA-SC for SI in Nigeria, using an implementation science framework.

Between 2021 and 2023, we conducted N=141 interviews with providers offering DMPA-SC for SI, and N=129 interviews with their clients using DMPA-SC for SI in Lagos, Enugu and Plateau States. Using Proctor *et al’s* implementation science framework, we noted observations for each interview question, extracted related quotes, and coded observations and quotes by implementation outcome (acceptability, appropriateness, feasibility, fidelity, cost, efficiency, safety, client-centredness and adoption).

Among clients, learning about DMPA-SC and SI from social network members facilitated *acceptability* and *adoption* of the method. Clients reported that provider outreach was appropriate for contraceptive information. However, providers desired support to mitigate their own out-of-pocket *costs* and enhance the *feasibility* of outreach. Occasionally, providers used clients’ age or education to decide whether they could self-inject independently, rather than clients’ ability to perform SI procedures, limiting *client-centredness*. Many providers felt their *fidelity* to SI provision protocols could improve with refresher trainings on the latest guidelines around offering SI. Clients indicated that proactive follow-up support from providers for continued SI and side effect management was *appropriate* and desired; providers concurred with offering such support.

Findings suggest that programme scale-up efforts should prioritise: (1) leveraging peer support or social networks to facilitate *acceptability* of DMPA-SC for SI among clients, (2) improving access to training aids to ensure *fidelity* to protocols and facilitate *adoption* among clients and providers, (3) emphasising shared decision-making in judgement-free client trainings to encourage *client-centredness,* and (4) investing in models for proactive follow-up support to improve *feasibility* of continuation for clients’ desired length of time.

**WHAT IS ALREADY KNOWN ON THIS TOPIC**

- Formative and pilot studies across low- and middle-income countries have demonstrated the feasibility and acceptability of implementing programmes to train and offer subcutaneous depot medroxyprogesterone acetate (DMPA-SC). This injectable contraceptive method is characterised by a small needle and prefilled syringe system that can be self-injected by women.

**WHAT THIS STUDY ADDS**

- Our study contributes three main innovations to the field: (1) employing an implementation science lens, (2) focusing on scale-up and sustainability, and (3) examining the entire self-injection (SI) journey including awareness and demand generation, training, and follow-up and disposal practices. These contributions facilitated recommendations for programme improvement during scale-up of DMPA-SC for SI programmes, to better meet the needs and preferences of providers and clients.

**HOW THIS STUDY MIGHT AFFECT RESEARCH, POLICY OR PRACTICE**

- We identified several possible programme improvements, including leveraging peer support to boost awareness of the options for SI of DMPA-SC, providing refresher trainings to providers and follow-up support to clients for better provider–client interactions, and ensuring that actual service provision emphasises that willingness, comfort and adherence to SI protocols are sufficient for unsupervised SI.

## Introduction

 Contraceptive service provision and access are subject to myriad social and structural challenges.^1 2^ Globally, pronatalist and patriarchal norms limit women’s sexual and reproductive health (SRH) decision-making power and their ability to access SRH services.^3 4^ Simultaneously, procurement and supply chain issues^5^ preclude healthcare providers from being able to offer women the full range of contraceptive methods. Innovations and service delivery models that mitigate these social and structural barriers are critical to ensuring that SRH services meet people’s needs.^6^ One such innovation, subcutaneous depot medroxyprogesterone acetate (DMPA-SC), an injectable contraceptive method with a small needle and prefilled syringe system, has been approved for self-injection (SI) by women. The option for SI is an important addition to the self-care ecosystem, comprising medications and diagnostic tools that can be used outside of the formal health system.^7^ By reducing the need for facility visits and placing control of contraceptive use in women’s hands, this option has the potential to increase women’s agency and reduce the burden on stressed healthcare systems.^8^

 Research has shown that SI of DMPA-SC is acceptable and feasible in low- and middle-income countries (LMICs).^9^,^15^ Research also shows that, despite experiencing some initial fear, women are able to successfully self-inject.^16^ The option to self-inject DMPA-SC could give women more control of their contraceptive decisions and behaviours, including using contraception covertly.^11^ Providers offering DMPA-SC for SI have expressed that the option for SI is acceptable for a wide range of clients.^9 10^ While acknowledging clients’ initial fear and the extra time needed for counselling and training them on proper SI procedures,^16^ providers recognise the potential long-term benefits of this option, including reducing their workload. However, implementation and scale-up of programmes offering DMPA-SC for SI face many challenges.^17^ To maximise the potential of this self-care intervention to meet providers’ and clients’ needs, employing an implementation science lens is crucial.^18^

 Nigeria was one of the first countries in Africa to introduce DMPA-SC, demonstrating government commitment to improving contraceptive service provision by strengthening the contraceptive supply chain and mitigating norms that hinder SRH access.^19^ Part of this effort involved the introduction of DMPA-SC in Nigeria in 2014 followed by an Accelerated Introduction and Scale-up Plan in 2017.^6 20^ Initial pilot programmes allowing only provider-administered DMPA-SC provision in limited geographies were progressively expanded across 10 states from 2017 to 2021 to allow private pharmacies and patent and proprietary medicine vendors (PPMVs) and public service delivery points (SDPs) to offer the method. Guidelines for enabling SI of DMPA-SC^21^ soon followed and were incorporated into implementation^19^ of pilot projects that offered SI training and counselling. Now, the offering of DMPA-SC for SI has been scaled to private SDPs in an additional eight states and to public SDPs in four new states. Programmes are implemented by two non-governmental organisations following national guidelines,^21^ which originally specified a multistep process for clients to learn independent SI: (1) providers train clients on SI and administer the first dose, (2) providers retrain clients and observe clients self-inject the second dose (about 3 months later), and (3) providers dispense units for unsupervised SI for the third and subsequent doses to clients who have successfully self-injected.

 As programmes offering DMPA-SC for SI are similarly underway in many LMICs, it is critical to understand what works to optimise scale-up and ensure the hypothesised benefits are realised. A comprehensive implementation focus is needed to inform contraceptive service scaling that centres women’s informed choice across method options, including DMPA-SC and SI. Researchers have highlighted the need for focused implementation research studies to develop contextually specific DMPA-SC for SI programmes,^22^ especially to inform scale-up.^17^ Embedding implementation research into contraceptive initiatives can help ensure they are adapted to meet clients’ and providers’ needs. Guided by the implementation science framework by Proctor *et al*,^23^ covering acceptability, appropriateness, feasibility, fidelity, cost, efficiency, safety, client-centredness and adoption, we conducted in-depth interviews (IDIs) with providers of DMPA-SC and their clients in three Nigerian states (Lagos, Enugu and Plateau) between 2021 and 2023. Here, we present results from the exploration of providers’ and clients’ experiences with providing and receiving services, respectively, for DMPA-SC for SI in Nigeria.

## Methods

**Figure 1:**
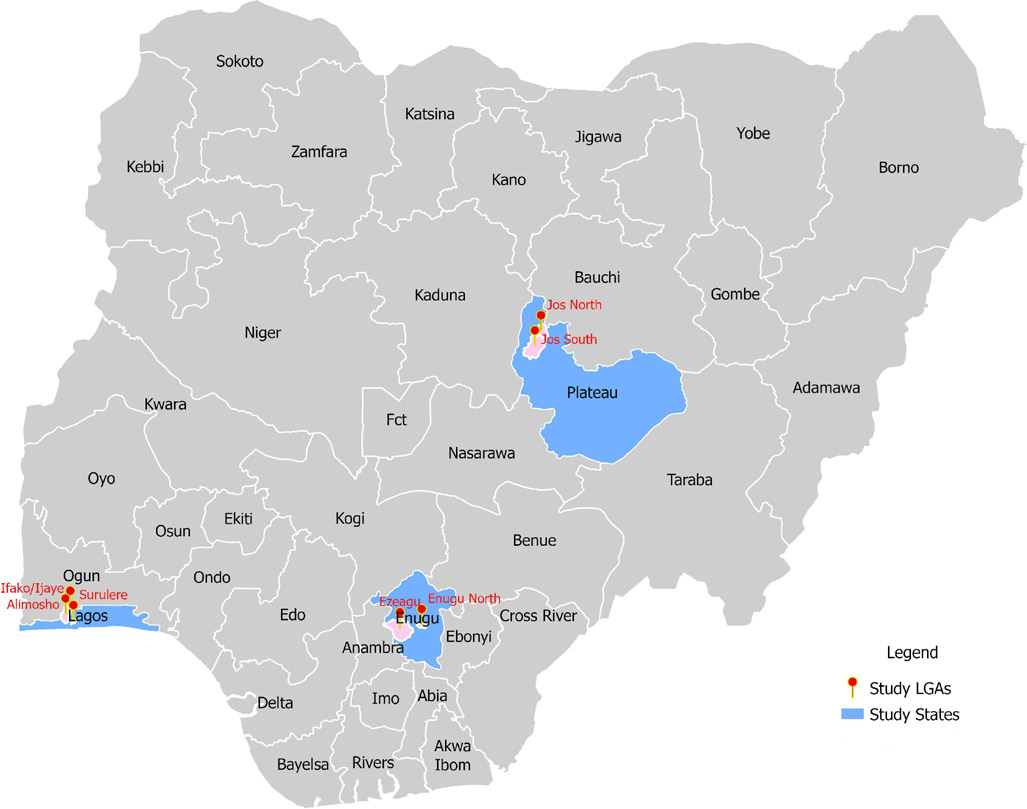
Map of local government area (LGA) study sites in Nigeria.

This qualitative descriptive study was one component of broader research on the implementation of DMPA-SC for SI in Nigeria, Kenya, Malawi and Uganda.^24^ Per methods described elsewhere,^25 26^ local government areas within three states in Nigeria were identified for this implementation research study: three in Lagos (South West region), two in Plateau (North Central region) and three in Enugu (South East region) (figure 1). These three states were selected from those in which implementing partner programmes were delivering DMPA-SC in the public sector and where expansion to the private sector was expected. Given Nigeria’s diversity, we prioritised selecting states that represent a range of geographic, socioeconomic and cultural environments balanced with accessibility of the state for data collection. Compared with Enugu, Plateau State is more rural, with earlier age at marriage for women, lower percentage of births at a facility, but higher percentages of family planning (FP) users obtaining their method from government facilities. Lagos is the most urban state where piloting of DMPA-SC for SI provision at private SDPs was conducted. Reflecting the total-market approach (i.e., implementation across public and private sectors)^19^ to DMPA-SC for SI implementation in Nigeria, public (public health clinics; PHCs) and private sector (clinics, pharmacies, chemist/PPMV stores) SDPs were sampled. From these SDPs, we interviewed providers trained to offer DMPA-SC for SI (N=141) and women (clients) who had self-injected DMPA-SC (N=119). Nigerian researchers fluent in English and local languages Igbo, Hausa, and Yoruba completed a 3-day training on conducting IDIs, including comprehensive review of the semistructured interview guide. Interviews were conducted from February 2021 to November 2023, to capture providers’ and clients’ perspectives on the implementation of DMPA-SC for SI programmes.

## Recruitment

### Provider IDIs

**Figure 2:**
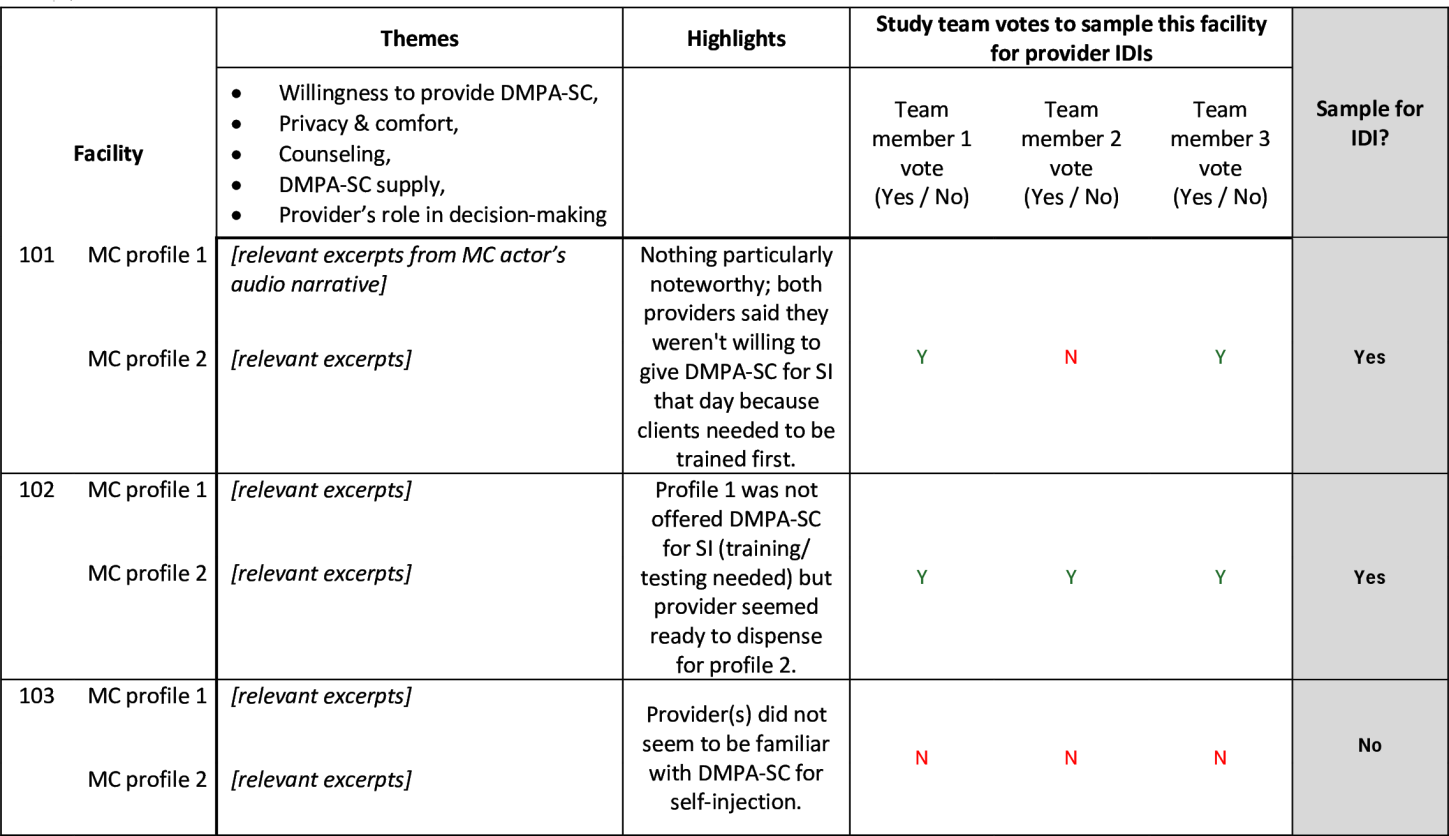
Template example for in-depth interview sampling from mystery client audio narratives. DMPA-SC, subcutaneous depot medroxyprogesterone acetate; SI, self-injection; MC, mystery client; IDI, in-depth interview. Details of the MC interaction were extracted from transcribed audio narratives in the ‘Themes’ column. Using the extracted excerpts, study team members summarized findings in the ‘Highlights’ column. Study team members then independently voted on whether to sample the facility for provider IDIs. Repurposed from “Setbacks in the road to self-injection: a descriptive study of provider and mystery client reports on the DMPA-SC care-seeking experience in Nigeria” (Griffith et al)^28^

Public and private retail sector health providers were recruited for IDIs based on outcomes of simulated (‘mystery’) client visits to selected SDPs to assess contraceptive counselling quality and comprehensiveness; details of visits are documented elsewhere.^25 27^ Mystery client visits involve simulated clients trained by researchers to observe first-hand provider behaviours. This is a widely used method in health research to study provider behaviour in a way that minimises observation bias.^28^ Mystery client actors audio-recorded brief post-visit impressions, which we used to select SDPs from which to recruit providers. Outcomes of the visit, such as whether the mystery client received DMPA-SC, client privacy within the SDP and the provider’s role in contraceptive decision-making were extracted from the post-visit transcripts. Research team members then voted on whether to sample the facility for a provider IDI with preference towards SDPs where mystery clients of two different profiles (an older, married client and a younger, single client) reported contrasting experiences (figure 2). In each state, mystery client visits were conducted once to capture the experience of clients initiating DMPA-SC, and a second time (roughly 1 year later) to capture the experience of clients seeking to obtain additional doses to continue SI of DMPA-SC.^26^ Since the sampling frame was SDPs visited by mystery clients, some providers were interviewed two times (n=7 in Lagos, n=1 in Enugu, and n=8 in Plateau). As interviews explored different topics at the two time points, and new information was gained from the topics covered in each interview, data were not analysed differently based on whether a respondent was interviewed at one or at both timepoints. The reported sample size is the number of interviews completed, and not necessarily the number of unique providers interviewed. Selected SDPs represented diverse reports of counselling quality, including actor’s privacy/comfort level, provider’s counselling on SI of DMPA-SC, and provider’s role in contraceptive decision-making. To ensure a range of perspectives, we included pharmacists and PPMVs from private SDPs, and community health practitioners and nurses/midwives from both public and private SDPs. In Nigeria, private SDPs operate as retailers, selling contraceptive products for a profit, and some charge the client for provider services such as administering an injection. Public SDPs receive contraceptives from the government and generally provide them to clients at no cost. Some may charge clients for materials used such as gloves and pregnancy tests.

### Client IDIs

Clients were identified through provider records and word-of-mouth referrals. To minimise referral bias, we recruited across a range of SDPs; furthermore, using provider records enabled the recruitment of clients who had sought care in both public and private SDPs. We purposively sampled different client profiles, including unmarried, married, adolescent, student, and professional women. Eligible participants were sexually active women aged 15–45 years with experience self-injecting DMPA-SC.

### Consent

Multilingual study team members with prior experience conducting IDIs called selected SDPs or clients, described the study, and scheduled a time for in-person informed consent and interview with interested participants. At the in-person scheduled interview, participants read or were read the consent form and either signed the consent form or gave verbal consent (if in-person signature was not feasible).

## Data collection

After obtaining consent, interviews were conducted following a semistructured guide covering topics such as the provider’s/client’s perception of DMPA-SC for SI, DMPA-SC training experience, and how they typically interact with clients/providers. Provider interview guides included questions on general contraceptive counselling procedures and DMPA-SC for SI provision practices. Providers were asked to reflect on both their own training for offering DMPA-SC for SI and their procedures for training clients on how to use DMPA-SC for SI. Additionally, providers were presented with vignettes about two different client profiles (one younger, unmarried woman and one older, married woman). They were asked to describe how they would counsel these hypothetical clients on contraception and specifically, if they would be well suited to self-inject DMPA-SC. Clients were asked to share their experience learning about, deciding to try, being trained on, and using DMPA-SC for SI. This included questions about the counselling they received (both about contraception generally and DMPA-SC for SI specifically), how they were trained to self-inject, and what their initial SI experiences were like. They were prompted to reflect on their satisfaction with the process of getting and using DMPA-SC for SI.

Interviews were conducted in the language of participants’ choice (Igbo, Hausa, Yoruba or English) by Nigerian female researchers in private, convenient locations chosen by participants; interviews lasted 1 to 2 hours and were audio recorded and transcribed. To ensure confidentiality, interviewees were instructed not to mention any names or identify any facilities by name in the interview recording. Provider interviewees were not specifically informed about the mystery client visits that preceded the interview. Interviews conducted in Igbo, Hausa or Yoruba were directly translated to English during the transcription process. Each interview transcript was quality checked by a different Nigerian team member. Transcripts were deidentified before analysis, and audio recordings were destroyed after transcription.

## Data analysis

### Conceptual Framework

The success of innovative treatments, programmes, or services depends on their robust implementation. A core tenet of implementation science is the examination of what, why and how evidence-based innovations work to maximise the health impact of these innovations. Employing an implementation science lens necessitates evaluation of specific domains that serve as indicators of implementation success as precursors to treatment, programme, or service outcomes. In this study, we draw inspiration from the conceptual framework developed by Proctor *et al*^29^ of the separate but related nature of implementation domains, service outcomes and client outcomes. Specifically, we were interested in applying specific constructs of Proctor *et al*’s taxonomy of implementation and service domains,^23^ including acceptability, appropriateness, feasibility, fidelity, cost, efficiency, safety, client-centredness and adoption to understand providers’ and clients’ experiences with DMPA-SC for SI.

### Analysis

**Figure 3:**
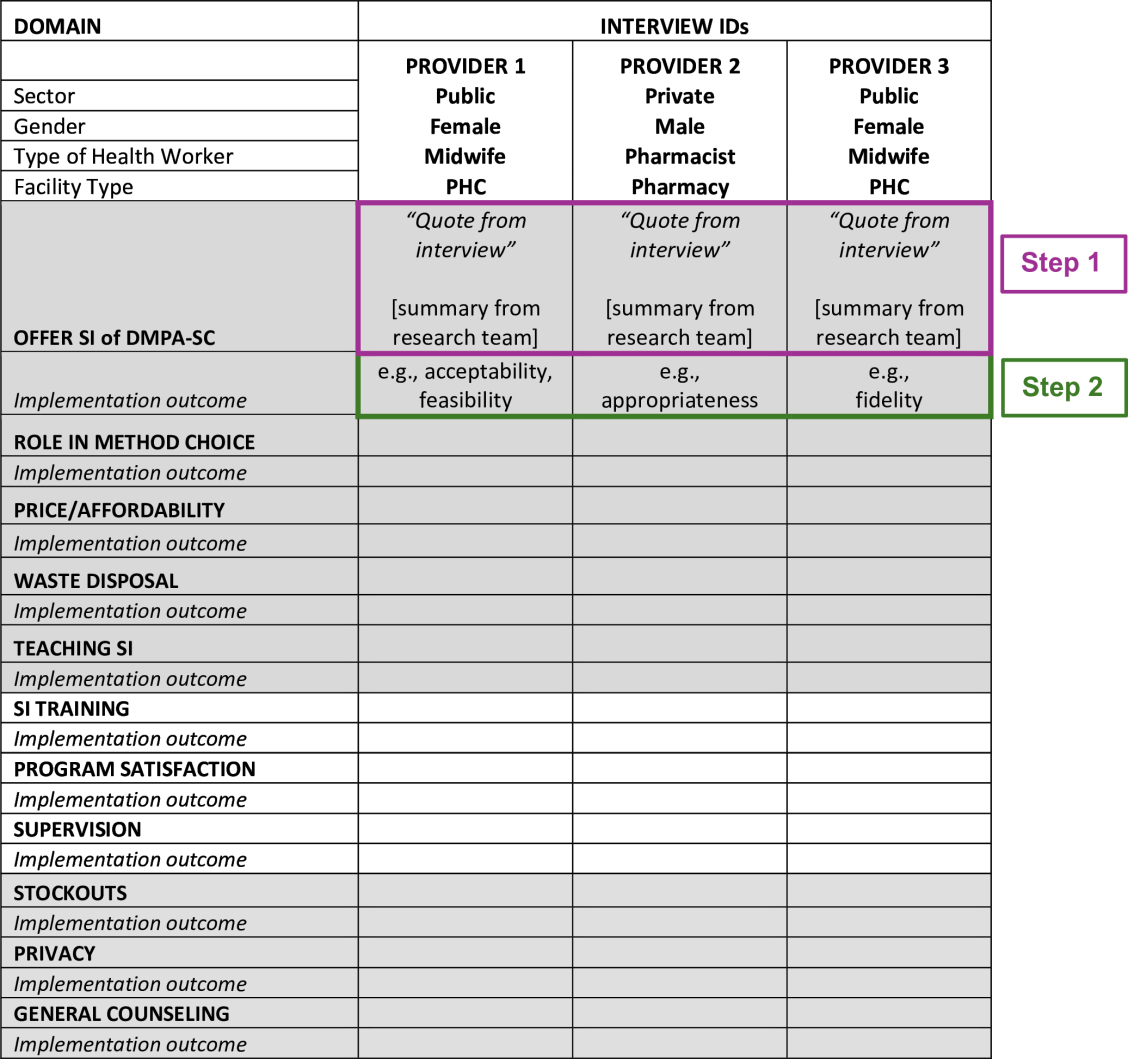
Provider in-depth interview analysis matrix example. DMPA-SC, subcutaneous depot medroxyprogesterone acetate; PHC, public health clinics; SI, self-injection. Step 1: Transcript data organized in matrix form by interview question: one column per interview, one row per question. Relevant quote and summary from research team recorded in matrix. Step 2: Implementation research domain(s) ‘assigned’ to findings where applicable.

We analysed transcripts through deductive coding by interview question and by implementation research outcome. To summarise data, we developed spreadsheet matrices with one column per transcript and one row per question (figure 3). Once transcription, translation, and deidentification were completed, Nigerian team members (including those who conducted the interviews) placed key observations and illustrative quotes from the transcripts in the appropriate cells of the matrix. Each matrix was reviewed by another team member for quality and completion. At this point, deidentified transcripts and preliminary analysis matrices were uploaded to a secure password-protected, cloud-based folder. Access to this folder could only be granted by principal investigators and was limited to key Nigerian and American team members. For analysis, team members accessed all transcripts and analysis matrices through these cloud-based folders from password-protected computers.

Next, we assigned between one and three of the most relevant implementation domains (adapted from Proctor *et al*, table 1)^23^ to summarise data for each question (i.e., we assigned domains to each row in the matrix). Five authors (Nigerian and American) participated in this phase of assigning implementation domains and all worked on the same 10 transcripts to establish intercoder reliability. After reaching consensus on how the implementation domains were defined and would be used, each analyst was assigned a unique set of 20 more transcripts to assign implementation domains. Again, we met to discuss and compile initial findings, and then assigned the remaining transcripts across the five analysts. With each successive batch of assigned transcripts, the analysts aimed to only add new or contradictory findings to the data we summarised in the preceding round.

**Table 1:** DMPA-SC for self-injection implementation research domains

Implementation outcome	Definition	Research questions
Acceptability	Satisfaction with various aspects of the innovation (content, complexity, comfort, delivery and credibility)	To what extent are clients and providers satisfied with SI services, training and delivery?
Appropriateness	Perceived fit; relevance; compatibility; suitability; usefulness; practicability	To what extent does SI fit clients’ and providers’ needs, preferences and practice?
Feasibility	Actual fit or utility; suitability for everyday use; practicability	When clients/providers reflect on SI use/counselling, respectively, how do they describe it addressing their needs and preferences?
Fidelity	Delivered as intended; adherence; integrity; quality of programme delivery	What does implementation look like in practice compared with the experience prescribed in national guidelines?
Cost	Marginal cost; cost-effectiveness; cost-benefit; opportunity	How do costs influence clients’/providers’ decisions to use/offer DMPA-SC for SI?
Efficiency	Time spent; time wanted; productivity	What are clients’ and providers’ perspectives on the time spent for use and provision of DMPA-SC for SI?
Safety	Safety and quality of service	To what extent is safety a concern for clients opting for DMPA-SC for SI and for providers counselling on/offering of DMPA-SC for SI?
Client-centredness	Extent to which services are responsive to clients’ needs, preferences and desires	To what extent do clients feel SI services are aligned with their needs?
Adoption	Uptake; utilisation; intention to try	Why are clients/providers choosing to use/offer DMPA-SC for SI?

Adapted from Proctor *et al.*’s framework.

DMPA-SC, subcutaneous depot medroxyprogesterone acetate; SI, self-injection.

**Figure 4:**
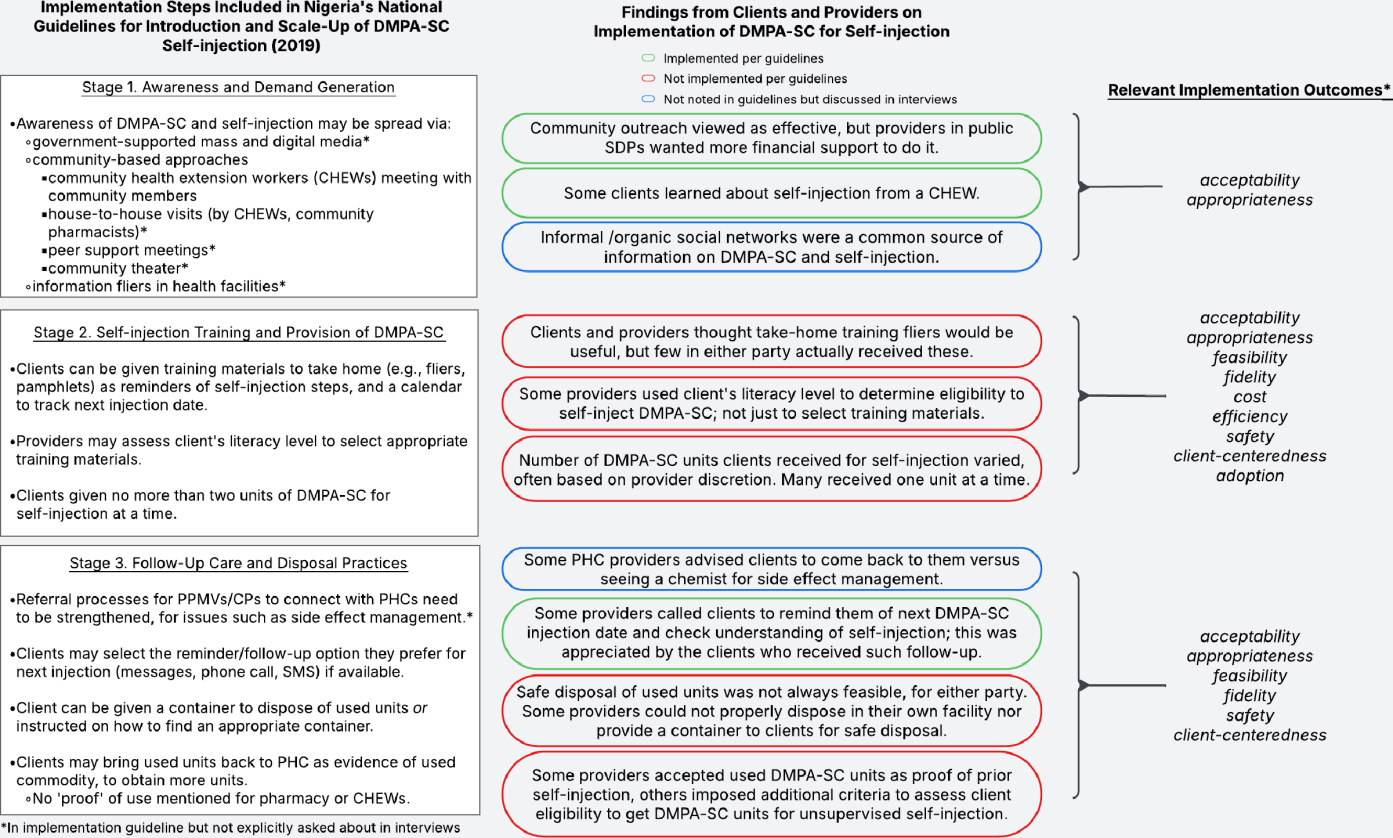
Comparing national introduction and scale-up guidelines with client and provider perspectives on DMPA-SC self-injection implementation. DMPA-SC, subcutaneous depot medroxyprogesterone acetate; PHCs, public health clinics; PPMVs, patent and proprietary medicine vendors; CPs, community pharmacists; SDP, service delivery points.*Adopted from Proctor et al^26^

Using the completed matrices, authors generated summary findings, organised by Proctor *et al*’s implementation research domains.^23^ Through this process, we aimed to use the implementation domains as an organisational tool for understanding and describing the findings across key stages of offering/trying SI of DMPA-SC: awareness and demand generation, training, and follow-up and disposal practices (figure 4). The process of assigning implementation domains to the findings was data driven, and as a result, some domains were more prevalent and more frequently assigned than others. We drew high-level findings within each stage of offering/trying SI of DMPA-SC, highlighting relevant implementation domains. To ensure findings were situated in the appropriate Nigerian social and structural context, the five analysis team members met to discuss, refine and finalise key findings and their interpretation.

## Results

Results include findings from IDIs with providers trained to offer self-injectable DMPA-SC (N=141) and clients who have self-injected DMPA-SC (N=119). Table 2 shows the characteristics of both client and provider participants. Findings are structured by key stages of offering/trying SI of DMPA-SC, mirroring those from national guidelines.^21^

**Table 2:** Client and provider participant characteristics

Client interviews (N=119)		
		n (%)
State	Lagos	44 (37.0)
	Enugu	45 (37.8)
	Plateau	30 (25.2)
Age	18–20	28 (23.5)
	21–30	37 (31.1)
	31–40	45 (37.8)
	41–45	9 (7.6)
Marital status	Married	93 (78.2)
Number of children	0	27 (22.7)
	1	16 (13.5)
	2+	76 (63.9)

Provider interviews (N=141)		
		n (%)
State	Lagos	51 (36.2)
	Enugu	45 (31.9)
	Plateau	45 (31.9)
Gender	Female	118 (83.7)
	Male	23 (16.3)
Type of provider	Community health practitioner (CHP)* in a PHC	48 (34.0)
	Pharmacist or PPMV†	36 (25.5)
	Nurse or midwife in a PHC	33 (23.4)
	CHP in private clinic/pharmacy/PPMV	10 (7.1)
	Nurse or midwife in private clinic/pharmacy/PPMV	8 (5.7)
	Officer in charge‡	6 (4.3)

* Community health practitioners (CHPs) are trained and licensed healthcare workers who deliver primary healthcare, particularly in rural areas, including health education, treatment of minor illnesses and FP services.^49 50^ CHPs include community-based distributors (CBDs), community health extension workers (CHEWs) and community outreach resource personnel.

† Patent and proprietary medicine vendors (PPMVs) sell pharmaceutical products for profit and are not formally trained in pharmacy.

‡ An officer in charge is responsible for managing the operations of a primary health facility. Depending on their qualification, they may be nurses, midwives, CHEWs, community health officer, etc.

FP, family planning; PHCs, public health clinics.

## Stage 1. Awareness and demand generation

### Many clients learned of DMPA-SC and the option for SI via social networks and provider outreach efforts

A majority of clients felt that both discussions with members of their social network, including friends, neighbours, and family members and discussion with providers conducting outreach were *appropriate* mechanisms for learning about DMPA-SC and the option for SI.

I have told people about it [DMPA-SC] […] one of them is using it now. [….] I would recommend it to as many as need it. (39-year-old client, Enugu)

I came to know that I can inject myself from a family planning provider; it was during an outreach service. (37-year-old client, Enugu)

Providers also viewed diffusion of messaging about the option for SI through clients’ social networks as *appropriate*; a couple of providers even mentioned that their client volume increased as a result.

When I am done with someone, she goes to tell another person [that I offer DMPA-SC for SI] and brings her to me. It has increased the number of clients that come for [DMPA-SC] for SI. (Female CHEW in a PHC, Enugu)

Providers in public SDPs commonly expressed a desire for more programme support to raise awareness of the option for SI and various access points in the communities they serve; this desire was occasionally mentioned by providers in private SDPs. A small number of providers specifically discussed the need for support to engage community leaders in raising awareness of contraceptive options.

Because they [community members] are familiar with us, they do not take us serious, but if people from the government come through the [community] leaders, they will give more attention to them. (Female CHEW in a PHC, Enugu)

[We could use support] to sensitize the community on the benefits of [DMPA-SC] and even how to give the injection. (Male CHEW in a private clinic, Plateau)

Another provider discussed needing financial support to conduct outreach to educate community members on FP, including on SI of DMPA-SC:

We spend money calling and visiting them [community members] […] In going to the community, it is my money that is involved…I need funding so that I can do it [educate more clients]. (Female Principal Nursing Officer in a PHC in Enugu)

### Accessing DMPA-SC for SI through multiple channels was acceptable and appropriate to clients

When deciding where to obtain DMPA-SC for initiating or continuing SI, many DMPA-SC users prioritised trust, comfort, and familiarity with a specific provider over other factors such as location of an SDP; this sentiment was expressed with regards to providers in both public and private SDPs.

[The provider] is very important to me because she is kind…Since I started using [DMPA-SC], I don’t visit [any other] hospital or clinic for it…I visit [that facility] because of that nurse. (37-year-old client, Plateau)

The thing with going to a hospital is you have to go where your heart accepts. This hospital is not so close to me, but […] I get along with the nurse there. (29-year-old client, Enugu)

Availability of DMPA-SC in both public and private sector channels in Nigeria, the ‘total market approach’, allowed some clients to obtain the method from their preferred provider or SDP. For example, a few clients preferred clinics over pharmacies or vice versa.

Maybe [I could also get DMPA-SC from] a hospital…or a pharmacy…I would like to get it there because a hospital is more organized [than the PHC I go to], and a pharmacy is too. (35-year-old client, Plateau)

I chose the [hospital] because I feel that they will know better about the drug and have better products than the pharmacy. (18-year-old client, Enugu)

Several clients noted that having multiple channels to choose from made access to DMPA-SC more *feasible*, especially when there were stockouts at certain locations.

If the day comes when it’s not [available] in the hospital, I’ll have to go to the chemist and get it myself. (43-year-old client, Plateau)

Sometimes if I go there [the clinic] and they don’t have it, she [the nurse] will just direct me to her friend who has a pharmacy. (24-year-old client, Plateau)

## Stage 2. Training for providers and clients on the provision and use of DMPA-SC for SI

### Training on offering DMPA-SC for SI and for learning to self-inject DMPA-SC for providers and clients, respectively, was generally viewed as acceptable and feasible

Training procedures were *acceptable* and *feasible* for a majority of clients and providers. However, clients commonly noted that being able to refer to visual materials, such as pamphlets with the SI steps, was helpful. While suggested in Ministry of Health guidelines,^21^ these materials were not readily available to many providers and clients interviewed.

They only showed us a book with pictures showing how to inject yourself… it was very useful to me. They also gave me flyers that I gave to other people as well. (39-year-old client, Lagos)

She didn’t show me any props or videos or pamphlets. She just talked with her mouth… Perhaps if they […] showed some pictures or charts, it would have helped me understand more. (30-year-old client, Enugu)

Providers echoed that having visual training aids for in-facility use and for clients to take home would have facilitated their adoption of the suggested training protocol.

Even the booklets that they drew step by step the way to self-inject, they should give it to us because it will help us to show the clients […]. (Female pharmacist in a PHC, Enugu)

If we can have a detailed manual on [DMPA-SC] SI, with a step-by-step procedure for the client to go home with, it will help. So that when they get home, and it is time for re-injection they will use it to watch the process again as they inject. (Female pharmacist, Enugu)

The stepwise SI initiation process required clients and providers to invest time in multiple in-facility injections before either party could reap the potential time-saving benefits of self-injectable contraception. However, clients rarely expressed dissatisfaction with *efficiency* or the time investment of learning SI. For example, when asked how the benefits compare to the effort required to learn how to self-inject, one client said:

There is nothing much, it is faster injecting myself, […] and it was worth the effort. (44-year-old client, Lagos)

In fact, most clients found the initiation process *acceptable* because they deferred to the provider on best practices.

I went back the second time to take the dose and after that, I asked her if she could give me to take home, she gave me and taught me how I could inject myself… I didn’t feel bad because I know she is qualified in her field, and she knows better. (19-year-old client, Enugu)

Most providers believed the training they received to offer DMPA-SC for SI was *acceptable* and *appropriate*. Two minor themes among providers were that their knowledge of contraception improved through the training, thereby increasing the feasibility of offering DMPA-SC for SI, and that their understanding of *client-centred* FP counselling also improved.

I really enjoyed the training because knowledge is power. When you have training on something, the knowledge is with you. (Female nurse in a PHC, Lagos)

It has exposed us better to family planning and we now pay more attention to it. We now do not just dispense or sell contraceptives, we can now provide professional advice and counseling to clients, so yes it has brought me value. (Male pharmacist, Lagos)

To ensure the continued *feasibility* of offering DMPA-SC for SI, most providers called for 'refresher trainings’, ranging in desired frequency from multiple times per year to annually, among those who specified. The requests often stemmed from appreciation for their initial training and a desire to stay current on the latest guidance for counselling on SI.

In the course of the training, we were told that Nigeria and the entire world are going towards self-care. Without that training, you may not understand the importance of that SI or self-care. (Female nurse in a PHC, Enugu)

I just want to learn more on [DMPA-SC] because it is new, and it is working. […] It [the training] made me work better and teach women on this new method. It has made them come more and more to this facility. (Female CHEW in a PHC, Enugu)

### The client-centredness of training clients and dispensing units for unsupervised SI of DMPA-SC was limited by providers’ relying on their discretion rather than the client’s demonstrated ability to safely self-inject

Providers’ perspectives revealed a variety of approaches to assess client eligibility for unsupervised SI. Providers commonly mentioned assessing clients’ education level, noting that it was less *feasible* to train clients with limited formal education to self-inject because they were perceived to have lower comprehension and confidence.

[…] they won't be able to do it. […] They don't have the education or the skills at all and the fear [of self-injecting] will be there. Some people might give themselves when their next dose is not even due, so we give them by ourselves. (Female CBD in PHC, Lagos)

It depends on how smart they are. In fact, there was one I gave to take home by the time she came back, she told me that, mummy, I couldn’t give it to myself that she wasted it. So, I started giving it to her by myself. (Female nurse in private clinic, Lagos)

Variation in providers’ assessments of clients’ eligibility for SI, along with inconsistencies in DMPA-SC dispensing practices, revealed inconsistent *fidelity* to national guidelines.^21^ However, clients still found these processes *acceptable*. Many clients received only one DMPA-SC unit to take home when seeking refills, while two units were recommended in the national guidelines.^21^ Despite the discrepancy, one unit was commonly *acceptable* to clients due to perceived limited *feasibility* of at-home storage (e.g., keeping needle away from children), concerns about confidentiality (e.g, from an unsupportive partner) or deference to the provider.

I was satisfied with one they gave me because I don’t have anywhere to hide it. (20-year-old client, Enugu)

I was given two sachets [units of DMPA-SC] […] I wouldn’t mind if more was given to me, but […] they [the providers] know better and have their reasons. (20-year-old client, Enugu)

Though many did not consider the possibility of receiving more than one unit, a few clients expressed that having more would have improved the *appropriateness* of the method choice.

I got just one. I followed her instructions since she is the nurse. If it were possible and allowed, I would have preferred more. Like two to avoid stories like missing my period or getting pregnant because I can’t get it quickly. (30-year-old client, Enugu)

Several providers did not find dispensing extra units of DMPA-SC for at-home storage to be *appropriate* or *feasible* for their clients. Similarly to clients’ reflections, providers were concerned about *safety* and liability, particularly with respect to clients with children or using contraception covertly.

[…] if I give you the one you will take home, can you keep it? Because if your husband finds out eventually, what happens there will be a problem. Maybe you are going to push the blame on me. (Female CHEW in PHC, Enugu)

There was a time a woman came and complained; we gave her one injection, one dose, and she didn’t store it well, and her children took it [to play with it]. That is very dangerous. […] those are some of the challenges. (Male CHEW in PHC, Plateau)

A few providers considered the distance clients travelled to the SDP, dispensing only one unit to those who lived nearby, and more to those who travelled further.

It depends on the distance of that person. […] If it is someone that lives very far […] and says that she doesn’t have any health centre close to her and that she wants to cover for the year […] I can give them three. People that live around here, I can give them one. (Female CHEW in PHC, Enugu)

Providers, including pharmacists, chemists and PPMVs, occasionally mentioned setting their own service fees and charging an administration fee in addition to fees for the product itself (DMPA-SC). These fees sometimes drove providers’ preferences to continue administering DMPA-SC, thus limiting clients’ access to self-care.

Is it not money that I am looking for? […] if the person comes to take the injection here, it will be better. The more profitable one is the one I inject them by myself. (Female pharmacist, Enugu)

### The potential efficiency associated with SI was hindered by transportation costs to obtain the method and stockouts

For clients who lived a greater distance from an SDP, access to DMPA-SC units was less *feasible*, especially when factoring in transportation costs.

I prefer going to [the provider’s] place because it’s not far from my place, considering the transport fare. If they want to give us someplace else and it’s far away, what about the transport fare? (39-year-old client, Lagos)

During the COVID period, […] [DMPA-SC] was not always available. We could go today and would be asked to come back another time. It was really stressful. (32-year-old client, Enugu)

The burden of stockouts was described by providers when reflecting on decisions about how much to charge (in private SDPs) or how many units to dispense (across public and private SDPs).

There was a time that we ran short of the commodity and had to go outside to get and that made us charge our clients [$0.13 USD] for it. Sometimes it’s rare for us to collect money or buy [DMPA-SC] outside […]. (Male CHEW in a private clinic, Plateau)

Usually, we give two or three [units]. Because if you pack it all and give them, others will come and may not get it, so we give them two [units] so that everyone will benefit. (Female CHEW in a PHC, Plateau)

## Stage 3. Follow-up care and disposal practices

### Clients found proactive follow-up support from providers for unsupervised SI acceptable and appropriate

The few clients who received follow-up support from providers appreciated it; while not explicitly required by the national guidelines,^21^ this support increased *acceptability* and *appropriateness* of clients continuing DMPA-SC, either self-injected or provider-administered. One provider-initiated innovation that enhanced *client-centredness* was follow-up phone calls. A couple of clients described receiving phone calls as reminders of their next injection date and found this follow-up strategy to improve *feasibility* of continuing the method.

She (the provider) does have the dates because if she calls, she usually records [the date]. Even if you forget, she will call you; that today you’re supposed to come and collect your injection after three months. (43-year-old client, Plateau)

I know that the next one is October even my provider calls me to remind me, so I don’t have to forget. (39-year-old client, Enugu)

One pharmacist said they call clients to assess understanding of SI in addition to reminding the client of their next injection date, though this practice was not common among private or public providers.

I follow up my clients on phone. When their time is due, I call them to explain to me the procedure they used to take the injection. I also call them at the due date for injection. (Female pharmacist, Enugu)

While most providers did not describe proactive follow-up with clients about side effects, several providers counselled clients to return to them if they had bothersome symptoms.

I will tell her that she should not go to the chemist to mix any tablet for any sign of side effect. She should call me. I will be in a position to know what to do for her. (Female CHEW in PHC, Enugu)

We give them the opportunity to ask questions while we answer and also encourage them to come back any time they have any problem or worries regarding any of the methods. (Female midwife in PHC, Lagos)

### While disposal of used DMPA-SC units was mostly safe and standardised in the clinical setting, at-home disposal practices varied widely and were not always carried out with fidelity to recommended guidelines

Providers commonly used government-supplied sharps boxes for in-facility disposal of used units and, when available, gave sharps containers to clients for at-home disposal. However, clients often improvised by storing used units in a plastic bag until they could be returned to a provider/SDP, burning used units, or throwing the unit in a latrine or nearby waterway.

Also, for the [self-injecting] client, I give them the sharp box too so when they are done with it, they keep it safe in the sharp box. When the one given to them is exhausted, […] we help them dispose of it in our safety box and return the sharp box to them. (Female CBD in PHC, Lagos)

They told me to throw it away and keep it out of the reach of children. When I use it, I usually throw it in the canal. (39-year-old client, Lagos)

Most providers disposed of used units in sharps boxes which were picked up by government entities for safe disposal. However, SDPs which did not have a government-provided hazardous waste pickup had to take their sharps box to a nearby PHC.

I have a safety box. There is really no challenge about it. If my safety box is about to be disposed, I will just take it to the nearest health center. It’s not far from here. (Female nurse/owner of private clinic, Lagos)

For most providers, disposing used units in a sharps box that was picked up periodically by a government entity was not an issue, but one superintendent pharmacist in Lagos noted difficulty with this process:

The challenge I am having is the sharp box is filled up but to deposit it… the primary health care center here that I know, I heard it’s no more there. (Female pharmacist, Lagos)

## Discussion

Guided by implementation science concepts from Proctor *et al*,^23^ we examined providers’ and clients’ perspectives on engaging with DMPA-SC for SI programmes in Nigeria, spanning the awareness/demand generation, training and administration/provision, and follow-up stages of offering/use DMPA-SC for SI. We found that themes of *appropriateness*, *acceptability*, *fidelity* and *adoption* were often present in providers’ and clients’ reflections on what was/was not working in the programmes being implemented for DMPA-SC for SI. Reflections on *feasibility*, *safety* and *client-centredness* were fewer but yielded important recommendations for future implementation practices, while *efficiency* and *cost* were rarely described. When comparing providers’ and clients’ experiences to the national guidelines for implementation, we identified some deviations from the guidelines that represent possible opportunities for improving implementation (figure 4). While implementation steps in the awareness and demand stage were generally implemented per the guidelines, there were several discrepancies between planned and actual implementation in the stage of training on and provision of DMPA-SC for SI. Factors such as DMPA-SC stockouts, client literacy level and provider concern for client safety inhibited provision of DMPA-SC for SI to interested clients. Within the follow-up stage, we found mixed adherence to the guidelines and opportunities for improving follow-up care and at-home DMPA-SC disposal practices.

During the awareness and demand generation phase, social networks were an *appropriate* and important source of information on DMPA-SC, and sometimes on the option to self-inject. Social marketing and behaviour change communication campaigns around FP have long been in place in Nigeria.^30^ However, there is less programmatic focus on leveraging more informal but highly trusted channels, such as friends, family and community members. This may be an important missed opportunity, especially as providers in our study echoed results from previous work^31^,^33^ showing that awareness and enthusiasm for new methods (including DMPA-SC for SI) in the communities they serve remain low. In addition to raising awareness, addressing social norms around the *acceptability* of contraception may also be critical, particularly since self-care options are newer. Studies in Benin,^34^ Cameroon,^35^ Kenya^36^ and Uganda^37^ have shown that when people perceive that contraceptive use is acceptable in their community, they are more likely to use contraception^10^ themselves. Strategies like network mapping and group dialogues^34^ or peer support groups^38 39^ could help create a normative environment supportive of people engaging in their desired contraceptive behaviours, including SI of DMPA-SC.

Training on offering and using DMPA-SC for SI was generally *acceptable*, but clients and providers noted areas for improvement. While the Ministry of Health guidelines^21^ recommend the use of visual job aids and marked calendars for clients, many providers lacked *fidelity* in using such aids, which were generally not available. Clients also reflected that increased availability of these materials would facilitate *adoption*. Another study in Nigeria showed that while 77% of interested clients were trained to self-inject, only 26% went on to *adopt* self-injectable DMPA-SC.^32^ While drop-offs on the journey to independent SI are expected, a lack of training tools may deter women from *adopting* the method. Providers felt having these tools and refresher trainings to stay updated on current protocols would enhance *feasibility* and *fidelity* of service provision. We found only one qualitative study in Malawi highlighting providers’ experiences after DMPA-SC for SI programme implementation.^10^ Our study highlights providers’ need for ongoing training, an aspect not fully detailed in Ministry guidelines^21^ but potentially necessary as SI programmes scale and guidelines evolve.

During the administration of DMPA-SC, providers exercised discretion in assessing client eligibility for independent SI. While guidelines^21^ allow clients to take home two doses after their second injection, providers commonly required additional supervised visits or limited the number of take-home doses. In our study, *client-centredness* in this form and client satisfaction with these aspects of dispensing were hard to gauge, as most deferred to their providers. While some clients may have been satisfied with this decision-making process, others may not have felt they had agency given that providers are perceived to hold superior knowledge and social status by virtue of their profession. Previous research in Nigeria has echoed this dynamic in which the provider is seen as the ultimate decision-maker.^40^ Client-centred service provision in Nigeria, especially with respect to self-care methods like DMPA-SC for SI, may necessitate efforts to emphasise the shared decision-making model given the reliance on client agency for use and adherence.^41^ This approach may be particularly critical for ensuring access among marginalised groups (e.g., those with less education, adolescents, etc.) who could benefit greatly from self-care methods.^42^

A prior study in Nigeria found that of 60 women trained to self-inject, only one was given additional doses to take home, highlighting that people may not be able to experience the full benefits of SI.^32^ Our findings suggest that provider hesitation to dispense stems from concerns that clients may not be able to safely inject and store units. It may also reflect personal biases, as previously documented,^43^ that manifest as unsanctioned, provider-imposed minimum age, education and parity eligibility requirements,^44^ and ultimately limit *client-centred* service provision. Research in Nigeria has shown that the effects of in-service training to address these restrictions are limited; only reduced marital status-related biases resulted from in-service training while age-related biases were unchanged.^44^ Providers bring their own personalities, beliefs and experiences to their client interactions. Thus, reducing provider bias goes beyond just addressing gaps in knowledge and requires more comprehensive social and behaviour change approaches.^2^ Additional research is needed to develop and test interventions to determine which approach (or combination of approaches) can be deployed at scale.

 Once clients begin independently self-injecting, models for waste disposal and follow-up support become crucial. The Nigerian Ministry of Health guidelines^21^ explicitly endorse storing used units in puncture-proof containers until they can be returned to a provider or picked up by a community health worker but waste disposal practices in our sample varied. Finding locally informed solutions that balance *safety* at the household level and *feasibility* at the health system level should be investigated (e.g., at-home pickup by community health workers or central drop-off locations in the community). At this stage, the quality and timeliness of follow-up support from providers also appeared *appropriate* and *acceptable* to the clients in our sample. While self-injecting DMPA-SC shows promise to increase contraceptive continuation among those who desire it, research has shown that missed reinjection windows, side effects and injection site reactions lead to discontinuation.^45 46^ As found in our sample, mechanisms to support independent SI such as reminders from providers for reinjection and referral systems for side effect management improve feasibility of continuation and should be prioritised during scale-up.

 Our results should be considered in light of several limitations. We had a select sample of those offering and using DMPA-SC for SI and results may not be generalisable to the general population of providers and FP clients. Specifically, providers were recruited from facilities where mystery client actors experienced varied contraceptive counselling quality. However, the providers interviewed were not necessarily the same providers actors visited, so it was not guaranteed that our data represent the range of quality delivered. Additionally, we could not interview every type of provider, limiting the roles and responsibilities represented in the data. We also engaged with a purposive sample, so the providers interviewed cannot be assumed to be representative of the broader provider population. However, in recent years, women comprise the majority of doctors and nurses in Nigeria,^47 48^ as reflected in our provider sample. There were limitations to our ability to tease apart differences in experience by provider type though we endeavoured to include different SDPs to surface differences in provider or client experience. In our analysis, few fundamental differences by provider type or SDP were found, so we presented perspectives in aggregate. Clients interviewed were women with experience self-injecting and were unique in having already surpassed barriers to SI access. We did not interview those who were turned away or denied access to DMPA-SC for SI and thus cannot speak to their experiences. Therefore, it is possible that our findings are skewed towards more favourable experiences. While we did implement purposive sampling, we could not ensure representation across age, parity and marital status dimensions, which did not allow us to speak to the full range of provider-imposed barriers women may face when accessing DMPA-SC for SI. Additionally, self-reported data from providers and clients may be influenced by social desirability bias. While there was no client or public involvement in the design of this study, we engaged closely with local implementing partner programmes to ensure our design and methods were relevant and appropriate for this implementation context. Furthermore, the nature of implementation research is such that stakeholder perspectives (i.e., from providers and clients) are deliberately included in programme and guideline modification. As such, results of our study have been disseminated to implementing partner programmes and the Ministry of Health.

## Conclusion

Our study is one of the first to use an implementation framework to examine providers’ and clients’ perspectives on delivery and use of DMPA-SC for SI. This approach was taken to better support integration of the SI option into the contraceptive method mix and to identify key areas for improvement as programmes are scaled across Nigeria and in other LMIC contexts. Our recommendations span the entire SI journey: leveraging peer support to boost awareness of the options for SI, providing refresher trainings to providers and follow-up support to clients for better provider–client interactions, and ensuring guidelines emphasise that willingness, comfort and adherence are sufficient for unsupervised SI. Findings suggest that national scale-up efforts should prioritise: (1) leveraging peer support or social networks to improve *acceptability* of DMPA-SC for SI, (2) increasing resources for providers and clients to have access to training aids to ensure *fidelity *to protocols and facilitate *adoption*, (3) emphasising shared decision-making in judgement-free provider trainings to encourage *client-*centredness, and (4) investing in models for proactive follow-up support to improve *feasibility* of continuation for clients’ desired length of time. These insights can support scaling SI programmes in a manner that meets provider and client needs.

## Supplementary material

10.1136/bmjgh-2024-018763 online supplemental file 1

## Data Availability

Data are available upon reasonable request.
