# Supplementary material for: Understanding clients’ and providers’ perspectives on the implementation of subcutaneous depot medroxyprogesterone acetate (DMPA-SC) for self-injection programming in Nigeria
Source: BMJ Glob Health. 2026 Feb 26;10(Suppl 6):e018763. doi: 10.1136/bmjgh-2024-018763 (PMC12958869; doi:10.1136/bmjgh-2024-018763)
Supplement: online supplemental file 1 [file bmjgh-10-Suppl_6-s001.docx]

### BMJ Global Health Author Reflexivity Statement

Adapted from Morton, B., Vercueil, A., Masekela, R., Heinz, E., Reimer, L., Saleh, S., Kalinga, C., Seekles, M., Biccard, B., Chakaya, J., Abimbola, S., Obasi, A. and Oriyo, N. (2022), Consensus statement on measures to promote equitable authorship in the publication of research from international partnerships. Anaesthesia, 77: 264-276. <https://doi.org/10.1111/anae.15597>

| **Study conceptualisation** | |
| --- | --- |
| 1. How does this study address local research and policy priorities? | Since 2017, the Government of Nigeria has committed to improving access to contraceptive methods through the public and private sector. The introduction and scale-up of self-injectable subcutaneous depomedroxyprogesterone acetate (DMPA-SC) is thus a priority of the Nigerian Ministry of Health. In this context, local implementing programs, researchers, and policymakers have sought evidence on the experiences and perceptions of DMPA-SC implementation. Relatedly, our study used an implementation science lens to explore providers’ and clients’ perspectives on implementation and understand what works. |
| 1. How were local researchers involved in study design? | This study was conducted through a partnership between UCSF and AkenaPlus Health, a Nigeria-based research consulting firm. Researchers from both organizations worked together on study design, meeting weekly to make joint decisions about site selection, sample size determinations, data collection modalities, and tool development. |
| **Research management** | |
| 1. How has funding been used to support the local research team(s)? | This study is funded by the Bill & Melinda Gates Foundation, Seattle, WA OPP1216593 (Co-PIs: Drs. Holt, Liu, and Omoluabi). Funding from this grant has provided research and salary support for the UCSF and AkenaPlus teams. |
| **Data acquisition and analysis** | |
| 1. How are research staff who conducted data collection acknowledged? | AkenaPlus research staff who led data collection are co-authors on this manuscript. |
| 1. How have members of the research partnership been provided with access to study data? | All co-authors on this manuscript have access to the data used to conduct this analysis. UCSF and AkenaPlus researchers have access to data collection tools, transcripts from in-depth interviews, and analysis tools/products. |
| 1. How were data used to develop analytical skills within the partnership? | Junior UCSF and AkenaPlus research staff received training in qualitative research, including in-depth interview data collection and analysis. Senior team members, including all three PIs and senior staff provided mentorship throughout the study, including on manuscript development and results dissemination. |
| **Data interpretation** | |
| 1. How have research partners collaborated in interpreting study data? | The analysis presented was conducted by UCSF and AkenaPlus researchers by collaborating on deductive coding by interview question and by implementation research outcome. We developed spreadsheet matrices that were used to generate summary findings. To ensure findings were situated in the appropriate Nigerian social and structural context, the five analyses team members (SC, MG, AJ, EH, AT) met to discuss, refine, and finalize key findings and their interpretation. Results were considered final after approval from all co-authors was obtained. |
| **Drafting and revising for intellectual content** | |
| 1. How were research partners supported to develop writing skills? | As part of the larger project funded by this grant, all junior staff, including those from UCSF and AkenaPlus have participated in writing workshops led by senior researchers. This has included an effort to pair junior team members with senior researchers for mentorship on an original analysis and manuscript. |
| 1. How will research products be shared to address local needs? | Research products from this study have been shared with local stakeholders on an ongoing basis through meetings with local program implementers and Ministry of Health officials. In addition to presenting and discussing findings, we have also developed and distributed results briefs that can be used for further dissemination. Local dissemination activities have been conducted to present and discuss results. |
| **Authorship** | |
| 1. How is the leadership, contribution and ownership of this work by LMIC researchers recognised within the authorship? | Co-authors on this manuscript include AkenPlus researchers who led data collection and analysis as well as the AkenaPlus PI. AJ and AT were part of the core data collection and analysis teams, II and CO led study design and data collection efforts, SD participated in data collection and analysis, and EO is the PI who has overseen all aspects of the study. |
| 1. How have early career researchers across the partnership been included within the authorship team? | Early career researchers at both UCSf and AkenaPlus Health have participated in trainings and workshops on qualitative research, data collection, and analysis. They have also received specific mentorship from senior researchers on abstract and manuscript development. |
| 1. How has gender balance been addressed within the authorship? | Most co-authors are women, which aligns with the fact that, due to the nature of contraception-focused research, many of our research participants are also women (all interviews with women participants were conducted by women researchers). |
| **Training** | |
| 1. How has the project contributed to training of LMIC researchers? | Our study’s research team members, including Nigeria-based AkenaPlus researchers, have participated in training on implementation research and qualitative methods. Early career researchers, again including Nigeria-based AkenaPlus researchers, also had the opportunity to participate in writing workshops and be matched with senior researchers as part of a writing mentorship program. |
| **Infrastructure** | |
| 1. How has the project contributed to improvements in local infrastructure? | Providers’ and clients’ perspectives elicited through the study have been shared with local stakeholders, including Ministry of Health officials. Findings from our work have been presented at local, regional, and international meetings accompanied by recommendations for program modifications. There are efforts underway to implement some of these recommendations. For example, informed by our work, the Ministry has reduced the number of in-person visits required for clients to be eligible to independently self-inject, facilitating improved efficiency and appropriateness. The ministry has also reduced the steps in the counselling process from 11 to 4. |
| **Governance** | |
| 1. What safeguarding procedures were used to protect local study participants and researchers? | The University of California, San Francisco Institutional Review Board (IRB # 20-32949, approved December 18, 2020) and the National Health Research Ethics Committee in Nigeria (study # NHREC/01/01/2007, approved August 25, 2020) approved these research activities. This includes all data collection modalities, recruitment methods, and consent procedures. Clients and providers were informed that participation was voluntary and that their responses would be kept private and confidential. Multilingual study team members with prior experience conducting IDIs called selected SDPs or clients, described the study, and scheduled a time for in-person informed consent and interview with interested participants. At the in-person scheduled interview, participants read or were read the consent form, and either signed the consent form or gave verbal consent (if in-person signature was not feasible). |
